# Noseband type and tightness level affect pressure on the horse's face at trot

**DOI:** 10.1111/evj.14420

**Published:** 2024-09-21

**Authors:** Russell MacKechnie‐Guire, Rachel Murray, Jane M. Williams, Jane Nixon, Mark Fisher, Diane Fisher, Vicki Walker, Hilary M. Clayton

**Affiliations:** ^1^ Equine Department Hartpury University Gloucester UK; ^2^ Rossdales Veterinary Surgeons Suffolk UK; ^3^ Buckingham Equine Vets Buckingham UK; ^4^ Woolcroft Equine Services Wisbech UK; ^5^ Department of Large Animal Clinical Sciences College of Veterinary Medicine, Michigan State University East Lansing Michigan USA

**Keywords:** bridle, horse, mandibular rami, nasal bones, pressure, welfare

## Abstract

**Background:**

Poor noseband adjustment could create high pressures that may risk pain or tissue damage.

**Objectives:**

To quantify sub‐noseband pressures dorsally over the nasal bone and ventrally over the mandibular rami for a Cavesson, Swedish (crank), Drop and Flash noseband at five tightness levels (2.0 to 0.0 finger equivalents).

**Study design:**

In vivo experiments.

**Methods:**

Eight high‐level dressage horses were ridden at the trot in a straight line by their usual riders. Two small pressure mats, attached to the noseband over the nasal bone and the mandibular rami, collected force (N) and pressure (kPa) data from four noseband types (Cavesson/Swedish/Flash/Drop) each adjusted to five tightness levels (2.0/1.5/1.0/0.5/0.0 finger equivalents) based on the use of a taper gauge. Noseband tightness and types were compared using Friedman's analyses with post hoc Wilcoxon tests (*p* ≤ 0.01).

**Results:**

Pressures (median and [25th and 75th percentiles]) and forces increased with tightness for all noseband types with higher mean pressures consistently recorded on the mandibles (Cavesson: 9.1 [5.0, 12.5] kPa, Swedish: 10.5 [6.3, 14.9] kPa, Flash: 8.0 [3.6, 15.2] kPa) than the nasal bones (Cavesson: 2.8 [1.1, 4.7] kPa, Swedish: 4.3 [3.1, 7.4] kPa, Flash: 4.9 [3.0, 7.3] kPa, *p* ≤ 0.002). None of the measured nasal pressures or forces differed significantly between tightness levels of 2.0 (1.6 [0.6, 3.6] kPa) and 1.5 fingers (2.9 [1.3, 4.1] kPa), but these values significantly increased from 1.0 (3.1 [1.5, 4.9] kPa), 0.5 (4.2 [2.3, 6.2] kPa), and 0.0 finger tightness (6.4 [3.8, 10.3] kPa) for most variables (*p* ≤ 0.004). No differences were found in mean/maximal nasal and mandibular pressures when fitted with a Cavesson or Swedish noseband.

**Main limitations:**

Behavioural and physiological parameters were not measured.

**Conclusions:**

Nasal and mandibular pressures increased with noseband tightness, with 1.0 finger laxity or less associated with significantly and incrementally higher pressures than 1.5 or 2.0 finger tightness.

## 1 INTRODUCTION

Bridle‐horse interaction is complex, with parts positioned near anatomical prominences, which could potentially create focal higher pressures. Nosebands have been used to stabilise bridles for hundreds of years.^1^ However, concerns about interactions between nosebands and the head have been raised, particularly the type^2^, ^3^, ^4^ and tightness^5^, ^6^, ^7^ of nosebands, so quantitative data are needed to support evidence informed noseband use to protect the welfare of ridden horses.

In trot, noseband pressures are cyclical (unpublished data from Murray et al., 2015), with maximum pressure occurring during early stance.^8^ Horses fitted with a Swedish (also referred to as a crank) noseband, with adaptations to reduce pressure, altered limb kinematics, providing evidence that noseband pressure influences locomotion.^8^ Pressure is highest over bony protuberances and shows an inverse relationship with the radius of curvature, which may mean that mandible pressures may be greater than nasal pressures.^5^ It has been shown that intermittent high pressures occur over the lateral margins of the nasal bone.^8^ High pressures over the mandibular rami have also been estimated based on mathematical calculation,^5^ reinforcing the need to consider the entire noseband. A range of noseband types are available. In equestrian sports, Cavesson and Flash nosebands are the most widely used,^6^, ^9^, ^10^, ^11^ with greater use of the Swedish (crank) noseband in dressage compared with show jumping.^7^ However, there has been little investigation of pressure patterns under different noseband types and how these are affected by tightness.

Noseband tightness has been raised as a welfare concern.^12^, ^13^ It has traditionally been recommended that two human fingers should fit between a correctly adjusted noseband and the horse's face.^14^ Despite this guidance, in a survey of horses competing in performance hunters, dressage and eventing competitions from 2013 to 2016, 44% had the noseband tightened to zero fingers, and only 7% complied with two‐finger recommendations.^10^ In 1529 of 3143 (49%) horses at Danish competitions where noseband tightness was measured, the noseband was tightened to less than 2 cm, determined by a modified noseband tightness measuring multi‐tool providing linear separation between the noseband and dorsal nasal bone.^6^ In addition, the results showed an association between a tighter upper noseband strap and the prevalence of ulceration at the lip commissures.^6^ More recent surveys have shown that 70% of Canadian sports horses^15^ and 59% of Dutch competition horses^7^ had nosebands adjusted to admit two fingers. To rationally guide practices that ensure welfare is not compromised, more information is needed on the relationships between noseband tightness and pressures.

The objectives of this study were, therefore, to quantify sub‐noseband pressures located dorsally over the nasal bone and ventrally over the mandibular rami for a Cavesson, Swedish (crank), Dropped (from here on referred to as a Drop) and Flash noseband at five tightness levels (2.0, 1.5, 1.0, 0.5, 0.0 finger equivalents). We hypothesised that (1) sub‐noseband pressures would increase with noseband tightness, (2) sub‐noseband pressures would differ between noseband types and (3) mandibular pressures would exceed nasal pressures.

## 2 MATERIALS AND METHODS

An a priori sample size calculation (G*Power) was performed using mean and standard deviation for paired differences for peak pressure data collected beneath the noseband in Murray et al.^8^ This estimated that for a sample size of eight horses, the study's power would be 0.90.

### 2.1 Horses

Eight high‐level dressage horses competing at Prix St. George to Grand Prix with (mean ± SD) height at the withers: 1.74 ± 0.07 m, body mass: 603 ± 24 kg and age: 12 ± 1 years were recruited. During their daily training horses were ridden in a Swedish (*n* = 4) or Cavesson (*n* = 4) noseband adjusted to a two‐finger equivalent tightness. Inclusion criteria included an absence of lameness and a full dental assessment by a qualified equine dental technician within 3 months of the study. On the day of data collection, horses were assessed visually by a veterinarian in walk and trot in a straight line on a firm, level surface to confirm an absence of lameness. Symmetry of the horses' gait was quantified using a validated sensor system^16^ (Equigait™).

### 2.2 Riders

Five experienced *Fédération Équestre Internationale* (FEI) competitive dressage riders (Prix St. George and above) were recruited with (mean ± SD) height: 1.70 ± 0.06 m and body mass: 75 ± 3 kg. Each horse was ridden by their usual rider. Three riders owned and rode two horses, and the remaining riders each rode one horse.

### 2.3 Bridles and nosebands

**FIGURE 1:**
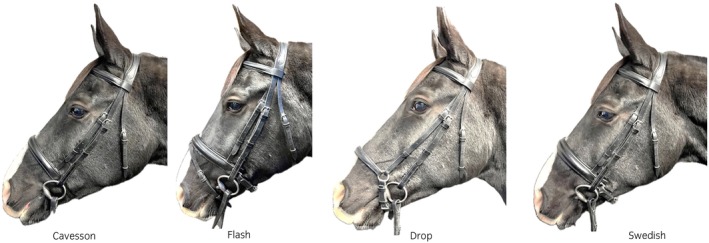
Left to right: Position and adjustment of Cavesson, Flash, Drop and Swedish (crank) nosebands.

Horsehead conformation was assessed by three Society of Master Saddlers‐qualified bridle fitters. A snaffle bridle with a padded headpiece, shaped around the caudal margin of the ears and including attachments for both cheek pieces and noseband, and a throat lash was fitted to each horse with the horse's usual bit, which was a double‐jointed snaffle (12.5–13.75 cm) in all cases. Holes were punched in the straps at 0.5 cm intervals for optimal fit. The nosebands used were (Figure 1):

- Cavesson: a simple padded (padding thickness: 4 mm) noseband featuring a single band encircling the nose 2–4 cm rostral to the facial crest, fastened with a buckle beneath the mandible.
- Swedish (crank) noseband (2): similar to a Cavesson with padding (4 mm) over the nasal bones. The strap ventral to the mandibular rami passed through two D rings and was adjusted to position its pad (thickness: 4 mm) over the mandibular rami.
- Drop noseband: the front of the noseband was positioned dorsal to the nasal bones, with its ventral strap passing rostral to the bit and sitting within the chin groove.
- Flash noseband: a combination of the Cavesson and a more rostral, thin strap that slid through a mid‐dorsal loop on the cavesson noseband and then passed rostral to the bit and within the chin groove.

### 2.4 Measurement systems

#### 2.4.1 Noseband tightness

The International Society of Equitation Science taper gauge^2^ was used to set noseband tightness. The gauge indicates one (18 mm wide × 11 mm depth) and two‐finger (40 mm wide × 16 mm depth) equivalent tightness levels. Horizontal reference lines were added at 50% of the distance between the 2.0 and 1.0 visual indicators and the end of the tool (0.0 finger tightness) to represent 0.5 (16 mm wide × 10 mm depth) and 1.5 (30 mm wide × 15 mm depth) finger‐equivalent tightness. The gauge was inserted mid‐dorsally beneath the noseband in a rostrocaudal direction until noseband tension resisted further movement. Zero finger tightness was defined as the inability to admit the taper gauge beneath the noseband and no compression of the soft tissues.

#### 2.4.2 Noseband pressure

**FIGURE 2:**
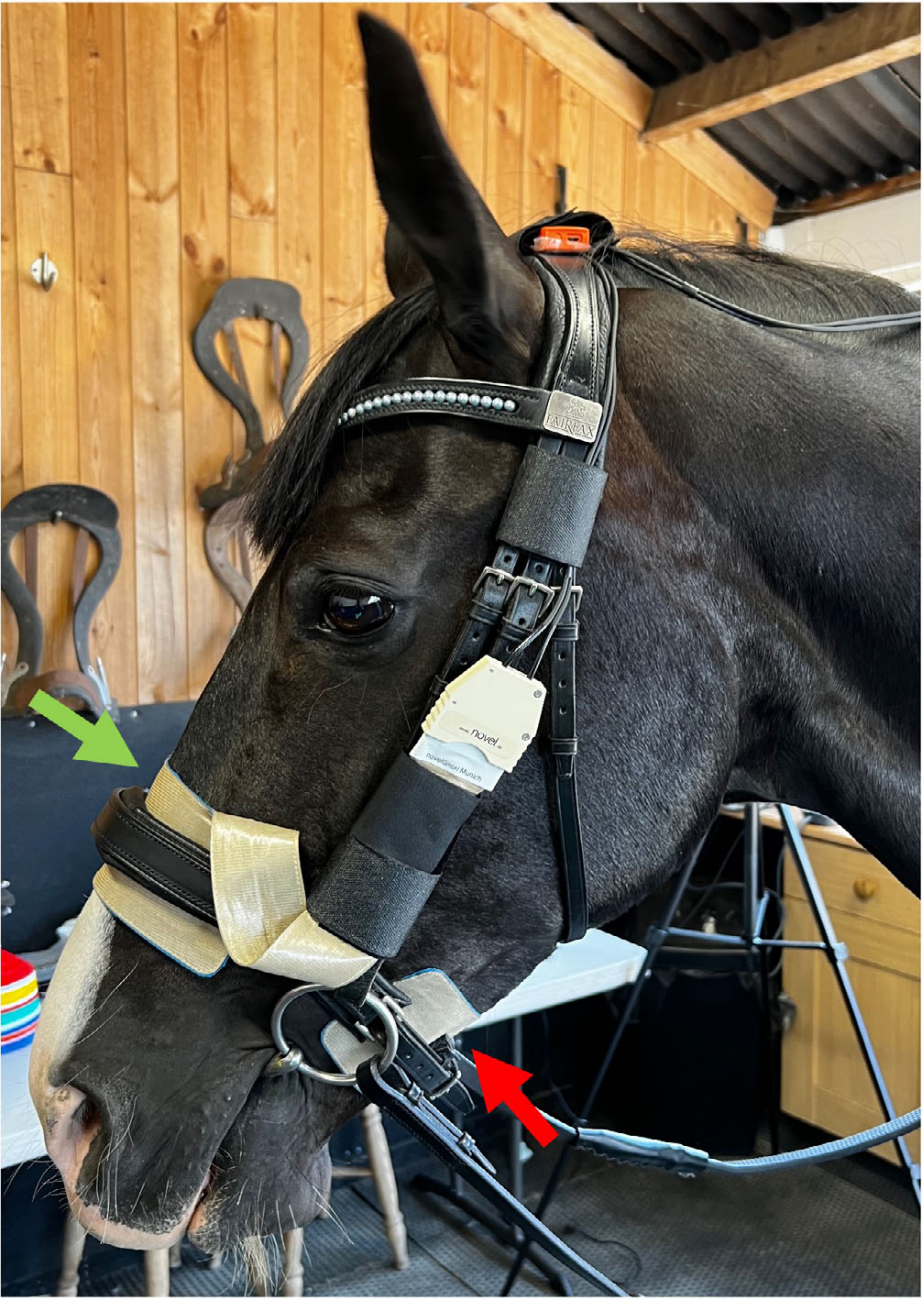
Positioning of the nasal (green arrow) and mandibular (red arrow) pressure mats beneath the noseband.

Two small pressure mats of sensor area 160 × 40 mm^2^, with sixty‐four 10 × 10 mm^2^ sensors/mat in a 16 × 4 configuration, and resolution 1 sensor/cm^2^ were used to measure pressure (Pliance; Novel GmbH™). These were positioned mid‐dorsally over the nasal bones and mid‐ventrally beneath the mandibular rami for each noseband. For the Flash noseband, measurements were made beneath the Cavesson part of the noseband. For the Drop noseband, only the nasal mat was used because the position of the ventral strap in the chin groove was not amenable to mat placement. Both mats were calibrated before the study within a range of 5–240 kPa. They were initialised to zero with the noseband loosely placed, then adjusted carefully, taking care that neither pressure mat moved. Cables from the mats were braided into the horse's mane and connected to a data logger attached to a saddle cloth (Figure 2). A 50 Hz video camera was synchronised with the pressure mats.

### 2.5 Study protocol

After fitting the pressure mats, a 10‐min rider prescribed warm‐up allowing familiarisation with the bridle was performed including walk, trot and canter on both reins. The noseband conditions (Cavesson, Flash, Drop, Swedish (crank)) were tested in random order. For each noseband, tightness increased sequentially in the order 2.0, 1.5, 1.0, 0.5 and 0.0 fingers as determined by the taper gauge. The 0.0 finger measurement was the first hole on the noseband strap at which it was not possible to insert the gauge.

Data collection took place along a 30 × 1.5 m runway in a 20 × 60 m arena with a wax‐coated surface. Horses entered the runway alternately from right and left directions and were ridden in a working trot (rising) with the front of the horse's face close to the vertical. Speed gates (Bower™) at each end of the runway transmitted the average speed of each trial wirelessly to a handheld unit. The first trial (two‐finger tightness) for each noseband type was used for comparison. Trials that differed by ±0.2 m/s were discarded^17^, ^18^; across all horses (mean ± SD) 3 ± 1 trials were repeated. A veterinarian observed the horses for behavioural indicators of pain or discomfort.^19^ No trials were terminated due to adverse behaviour or lameness. After completing data collection, the noseband was loosened to two‐finger equivalents, the horses were cooled down, then the equipment was removed.

### 2.6 Data processing

Ten consecutive trot strides per trial were analysed. Mean, maximal and minimal pressures (kPa) and total calculated force (N) were summed over all loaded sensors were determined on a stride‐by‐stride basis, exported to Signal View™ and filtered using a low pass 10 Hz filter. Preliminary analysis (see Section 2.7 below) indicated no significant differences in pressure or force variables when horses approached the runway on the left versus the right rein (all *p* > 0.05). Therefore, data for the two directions were pooled for further analysis.

### 2.7 Data analysis

The Kolmogorov–Smirnov test indicated that data distribution was non‐parametric, therefore, a Friedman two‐way ANOVA was used to compare pressures between noseband types and between tightness levels. When significant differences were found, post hoc Wilcoxon signed‐rank tests identified if differences occurred between noseband types and tightness. Differences between nasal and mandibular pressures (kPa) within horses were investigated using Wilcoxon signed‐rank tests. As this study included repeated measures, a Bonferroni adjustment was applied, therefore adjusting the significance level to *p* ≤ 0.01.

## 3 RESULTS

### 3.1 Effect of noseband type

**FIGURE 3:**
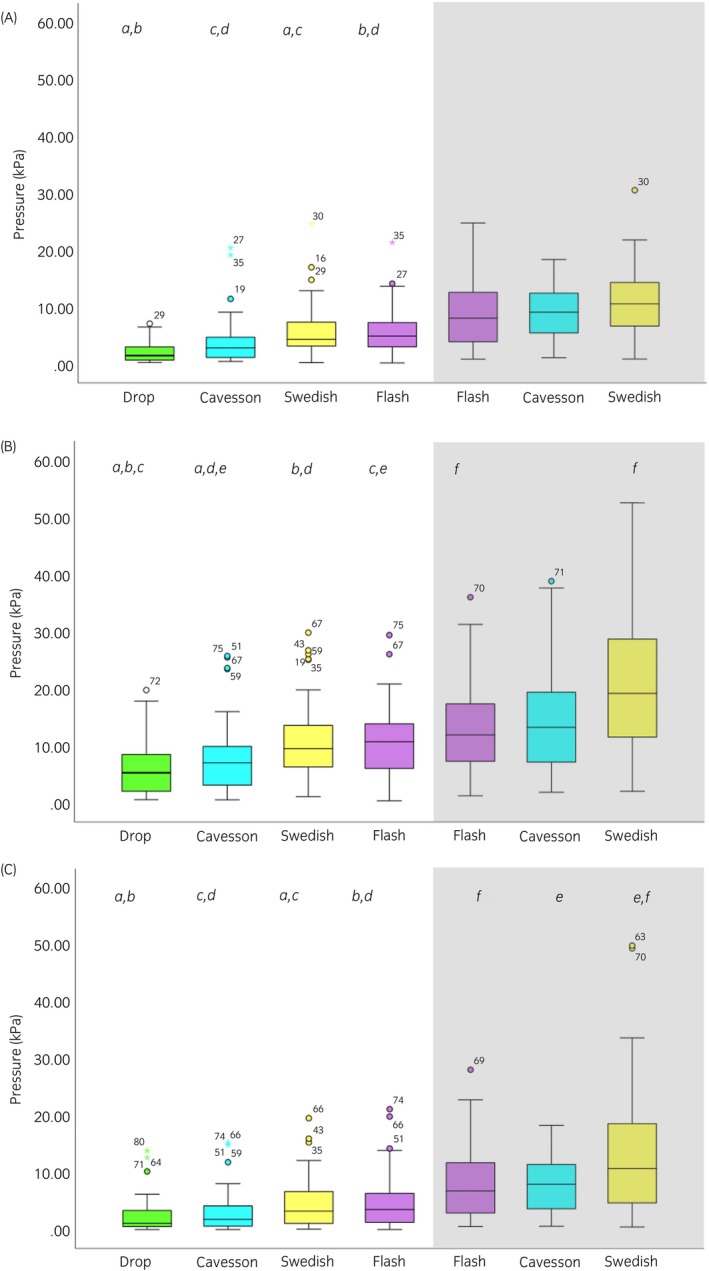
Boxplots illustrating (A) mean, (B) maximal and (C) minimal pressures (kPa) across noseband types for nasal and mandibular pressures (shaded area) when ridden in rising trot when fitted with a Drop (green), Cavesson (cyan), Swedish (yellow) and Flash (purple) noseband. The central line represents the median; the box represents the 25th and 75th percentile; and the whiskers represent the maxima and minima, not considered outliers. ° represents outliers and * represents extreme outliers. Superscripts [a–d (mean), a–f (maximal) and a–f (minimal)] represented by the same character indicate post hoc differences between two nosebands, values can be viewed in Table 1.

Across all noseband tightness levels, significant differences between noseband types were found for mean nasal pressure (*p* ≤ 0.001) and for maximal and minimal nasal and mandibular pressures (all *p* ≤ 0.001) (Table 1). For all noseband types, minimal, maximal and mean pressures were higher at the mandibular compared with the nasal site (all *p* ≤ 0.002). No differences in total force were found among noseband types (Table 1; Figure 3).

**TABLE 1:** Median and [25th and 75th] percentiles for nasal and mandibular mean, maximal and minimal pressure (kPa), and total force (N) across noseband types with comparisons between nasal and mandibular sites for 20 trot strides (*N* = 8 horses).

	Location	Cavesson	Swedish	Drop	Flash	*p* Value	Post hoc
Mean pressure (kPa)	Nasal	2.8 [1.1, 4.7]	4.3 [3.1, 7.4]	1.4 [0.6, 3.1]	4.9 [3.0, 7.3]	**<0.001**	Drop < Swedish, *p* = 0.001^a^ Drop < Flash, *p* = 0.001^b^ Cavesson < Swedish, *p* = 0.006^c^ Cavesson < Flash, *p* = 0.003^d^
	Mandibular	9.1 [5.0, 12.5]	10.5 [6.3, 14.9]	–	8.0 [3.6, 15.2]	0.12	
	*p* Value	**<0.001**	**<0.001**		**0.002**		
Maximal pressure (kPa)	Nasal	7.0 [3.1, 10.0]	9.5 [6.2, 13.5]	5.3 [2.1, 8.4]	10.6 [5.9, 13.8]	**<0.001**	Drop < Cavesson, *p* ≤ 0.001^a^ Drop < Swedish, *p* ≤ 0.001^b^ Drop < Flash, *p* ≤ 0.001^c^ Cavesson < Swedish, *p* = 0.005^d^ Cavesson < Flash, *p* ≤ 0.001^e^
	Mandibular	13.2 [7.1, 19.3]	19.1 [11.2, 28.8]	–	11.9 [7.2, 17.8]	**<0.001**	Flash < Swedish, *p* ≤ 0.001^f^
	*p* Value	**<0.001**	**<0.001**		**0.001**		
Minimal pressure (kPa)	Nasal	1.8 [0.5, 4.4]	3.2 [1.0, 6.7]	1.1 [0.5, 3.3]	3.5 [1.2, 6.5]	**<0.001**	Drop < Swedish, *p* ≤ 0.001^a^ Drop < Flash, *p* ≤ 0.001^b^ Cavesson < Swedish, *p* = 0.004^c^ Cavesson < Flash, *p* ≤ 0.001^d^
	Mandibular	8.1 [3.6, 11.6]	10.9 [4.6, 18.5]	**–**	6.9 [2.9, 12.4]	**<0.001**	Cavesson < Swedish, *p* = 0.002^e^ Flash < Swedish, *p* = 0.004^f^
	*p* Value	**<0.001**	**<0.001**		**<0.001**		
Total force (N)	Nasal plus mandibular	80.9 [44.1, 104.6]	108.1 [47.6, 137.5]		80.4 [47.6, 137.5]	0.41	

*Note*: Bold values indicate significant differences (*p* ≤ 0.01) between noseband conditions, and between nasal and mandibular pressures. Shaded boxes indicate no statistical test performed, and ‘–’ indicates no data obtained. Superscripts [a–d (mean), a–f (maximal) and a–f (minimal)] represented by the same character indicate post hoc differences between two nosebands; boxplots can be viewed in Figure 3.

### 3.2 Effect of noseband tightness

**FIGURE 4:**
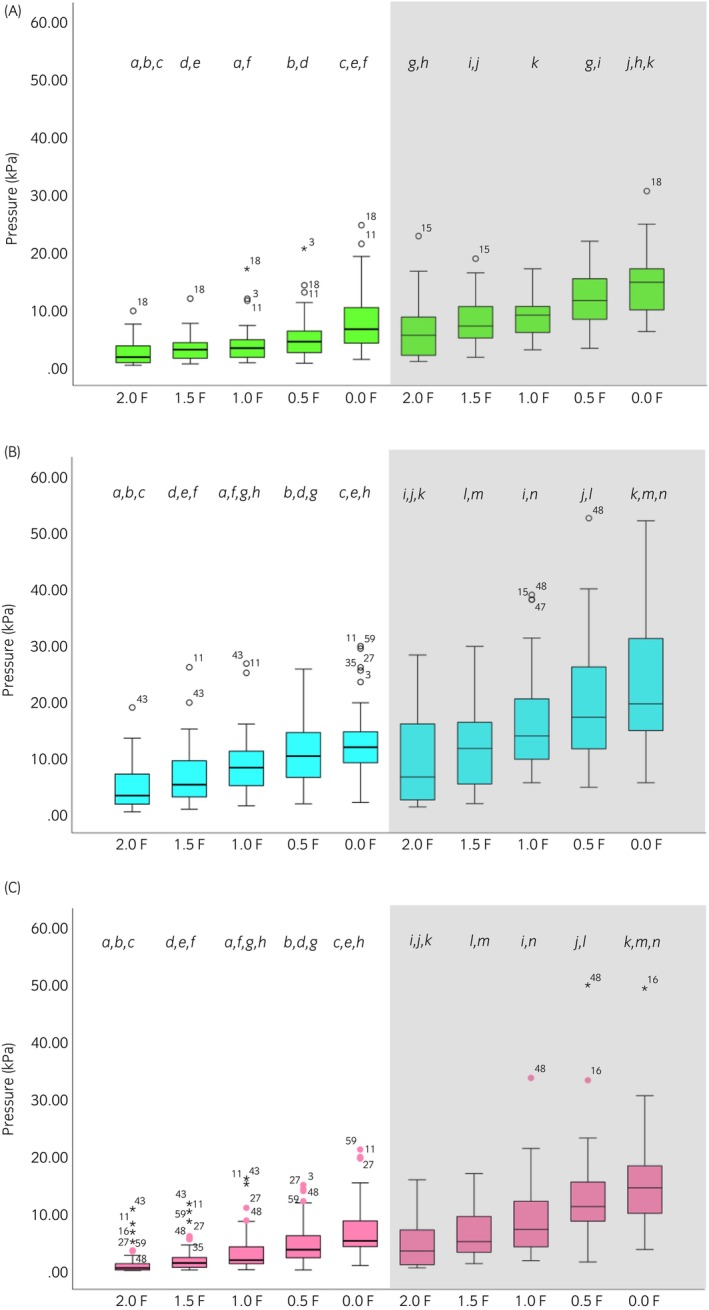
Boxplots illustrate (A) mean, (B) maximal and (C) minimal pressures when the noseband was adjusted from 2.0 fingers to 0.0 fingers for nasal and mandibular pressures. The shaded area represents mandibular pressures. The central line represents the median; the box represents the 25th and 75th percentile, and the whiskers represent the maxima and minima not considered outliers. ° represents outliers and * represents extreme outliers. Superscripts [a–k (mean), a–n (maximal) and a–n (minimal)] represented by the same character indicate post hoc differences between two nosebands; values can be viewed in Table 2.

Across noseband types, significant differences between noseband tightness levels were found for mean, maximal, and minimal nasal and mandibular pressures and total force (all *p* ≤ 0.001). Table 2 shows the post hoc analyses. For all tightness levels, mean, maximal, and minimal pressures were higher on the mandibles than on the nasal bones (all *p* ≤ 0.001). Total force differed significantly between noseband tightness levels (*p* ≤ 0.001) (Table 2; Figure 4).

**TABLE 2:** Median and [25th and 75th] percentiles for nasal and mandibular mean, maximal and minimal pressure (kPa), and total force (N) across noseband tightness and comparisons between nasal and mandibular sites for 20 trot strides (*N* = 8 horses).

	Location	2.0 Finger tightness	1.5 Finger tightness	1.0 Finger tightness	0.5 Finger tightness	0.0 finger tightness	*p* Value	Post hoc
Mean pressure (kPa)	Nasal	1.6 [0.6, 3.6]	2.9 [1.3, 4.1]	3.1 [1.5, 4.9]	4.2 [2.3, 6.2]	6.4 [3.8, 10.3]	**<0.001**	2.0 < 1.0 finger, *p* = 0.004^a^ 2.0 < 0.5 and 0 fingers, both *p* ≤ 0.001^b,c^ 1.5 < 0.5 fingers, *p* = 0.004^d^ 1.5 < 0.0 fingers, *p* ≤ 0.001^e^ 1.0 < 0.0 fingers, *p* ≤ 0.001^f^
	Mandibular	5.4 [1.7, 8.5]	7.0 [3.8, 10.9]	8.9 [5.4, 10.5]	11.4 [7.7, 15.2]	14.6 [9.2, 17.6]	**<0.001**	2.0 < 0.5 and 0 fingers, both *p* ≤ 0.001^g,h^ 1.5 < 0.5 fingers, *p* = 0.03^i^ 1.5 < 0.0 fingers, *p* ≤ 0.001^j^ 1.0 < 0.0 fingers, *p* = 0.006^k^
	*p* Value	**<0.001**	**<0.001**	**<0.001**	**<0.001**	**<0.001**		
Maximal pressure (kPa)	Nasal	3.2 [1.7, 7.1]	5.2 [2.9, 9.4]	8.2 [4.8, 11.2]	10.2 [6.4, 14.4]	11.8 [8.9, 14.7]	**<0.001**	2.0 < 1.0, 0.5 and 0.0 fingers, all *p* ≤ 0.001^a,b,c^ 1.5 < 0.5 and 0.0 fingers, both *p* ≤ 0.001^d,e^ 1.5 < 1.0 fingers, *p* = 0.01^f^ 1.0 < 0.5 fingers, *p* = 0.01^g^ 1.0 < 0.0 fingers, *p* ≤ 0.001^h^
	Mandibular	6.5 [2.5, 16.1]	11.6 [5.3, 16.6]	13.8 [9.6, 20.5]	17.1 [11.5, 26.1]	19.5 [14.6, 31.9]	**<0.001**	2.0 < 1.0, 0.5 and 0.0 fingers, all *p* ≤ 0.001^i,j,k^ 1.5 < 0.5 and 0.0 fingers, both *p* ≤ 0.001^l,m^ 1.0 < 0.0 fingers, *p* = 0.004^n^
	*p* Value	**<0.001**	**<0.001**	**<0.001**	**<0.001**	**<0.001**		
Minimal pressure (kPa)	Nasal	0.4 [0.1, 1.1]	1.2 [0.5, 2.2]	1.7 [1.1, 4.2]	3.6 [2.1, 6.1]	5.1 [4.1, 8.7]	**<0.001**	2.0 < 1.0, 0.5 and 0.0 fingers, all *p* ≤ 0.001^a,b,c^ 1.5 < 0.5 and 0.0 fingers, both *p* ≤ 0.001^d,e^ 1.5 < 1.0 finger, *p* = 0.01^f^ 1.0 < 0.5 fingers *p* = 0.01^g^ 1.0 < 0.0 fingers, *p* ≤ 0.001^h^
	Mandibular	3.3 [0.9, 7.1]	5.0 [3.1, 9.4]	7.1 [3.9, 12.7]	11.1 8.4, 15.5]	14.4 [9.9, 18.2]	**<0.001**	2.0 < 1.0, 0.5 and 0.0 fingers, all *p* ≤ 0.001^i,j,k^ 1.5 < 0.5 and 0.0 fingers, both *p* ≤ 0.001^l,m^ 1.0 < 0.0 fingers, *p* ≤ 0.001^n^
	*p* Value	**<0.001**	**<0.001**	**<0.001**	**<0.001**	**<0.001**		
Total force (N)	Total force nasal plus mandibular	43.7 [21.8, 89.9]	66.9 [48.4, 84.8]	80.3 [62.6, 111.9]	102.5 [87.8, 137.3]	149.2 [90.6189.7]	**<0.001**	2.0 < 0.5 and 0.0 fingers, both *p* ≤ 0.001 1.5 < 0.5 fingers, *p* = 0.01 1.0 < 0.0 fingers, *p* = 0.008

*Note*: Bold values indicate significant differences (*p* ≤ 0.01) between conditions, and nasal and mandibular pressures. Shaded boxes indicate no statistical test was performed. Superscripts [a–k (mean), a–n (maximal) and a–n (minimal)] represented by the same character indicate post hoc differences between two nosebands; boxplots can be viewed in Figure 4.

### 3.3 Nasal and mandibular pressures for four noseband types adjusted sequentially from 2.0 to 0.0 finger tightness equivalents

**FIGURE 5:**
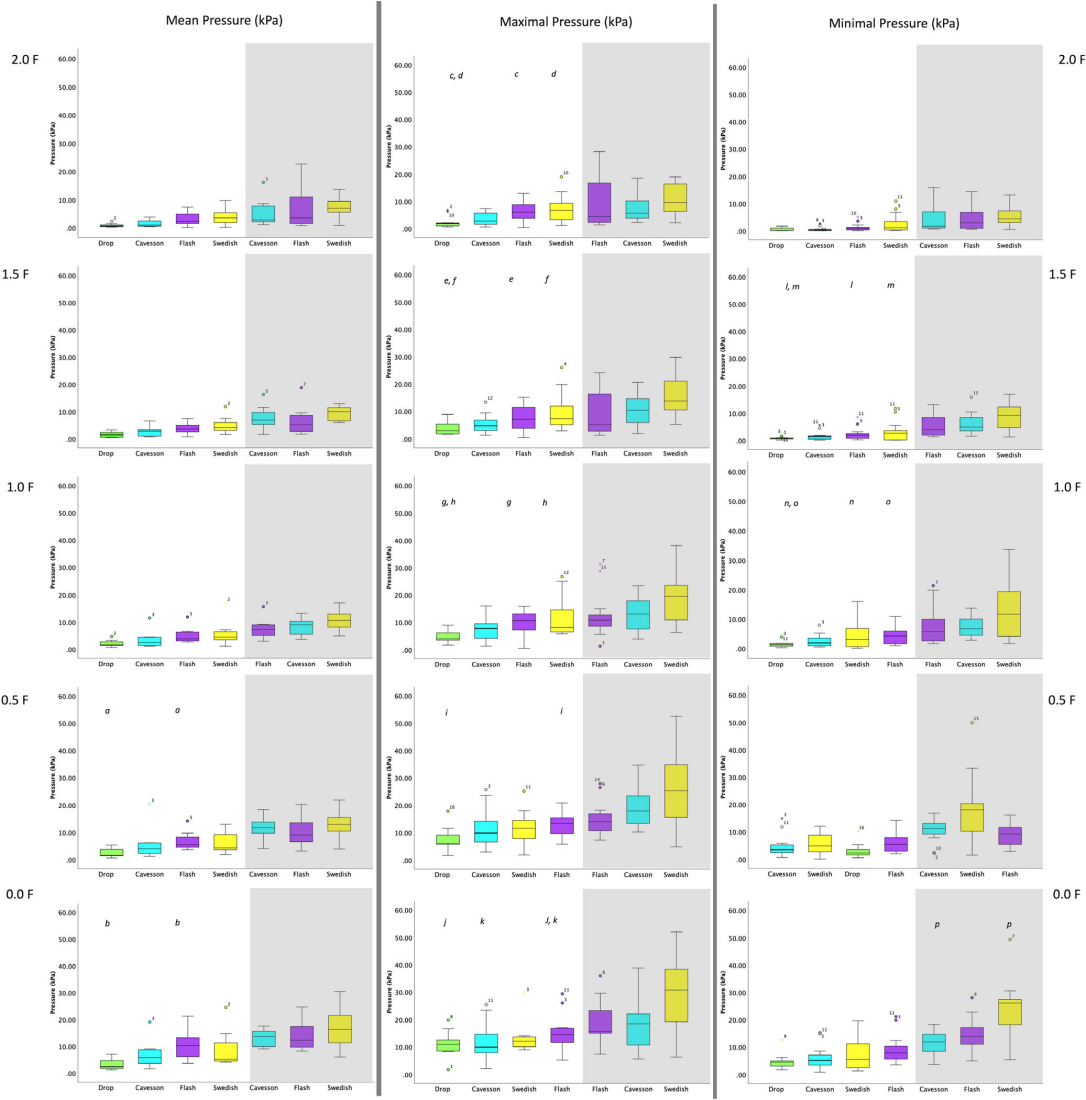
Boxplots illustrating mean (left column), maximal (middle column) and minimal (right column) pressures when the noseband was sequentially adjusted from 2.0 fingers (upper row) to 0.0 fingers (lower row) for nasal and mandibular pressures (shaded area) when ridden in rising trot when fitted with a Drop (green), Cavesson (cyan), Swedish (yellow) and Flash (purple) noseband. The central line represents the median; the box represents the 25th and 75th percentile, and the whiskers represent the maxima and minima, not considered outliers. ° represents outliers and * represents extreme outliers. Superscripts (a–p) represented by the same character indicate post hoc differences between two nosebands.

Differences in mean nasal pressure were found between noseband types when adjusted to 1.5, 0.5, and 0.0 finger tightness (*p* = 0.008, *p* = 0.01 and *p* = 0.006, respectively), with the Drop noseband having lower mean nasal pressure values than the Flash at 0.5 and 0 fingers *p* = 0.01 and *p* = 0.005, respectively. No significant differences were found between the remaining noseband types or tightness conditions (*p* ≥ 0.03). At 1.5 and 0.0 finger tightness levels, for the Cavesson and Swedish (crank) noseband, mean pressures were higher for the mandible compared with nasal pressures (all *p* ≤ 0.01). No differences were found between the mean mandibular and nasal pressures for the flash noseband (*p* > 0.16) (Table 3; Figure 5).

**TABLE 3:** Median and [25th and 75th] percentiles for nasal and mandibular mean, maximal and minimal pressure (kPa) for each noseband tightness level and comparisons between nasal and mandibular sites for 20 trot strides (*N* = 8 horses) for the Cavesson, Swedish, Drop and Flash noseband.

Tightness (fingers) variable	Location	Cavesson	Swedish	Drop	Flash	*p* Value	Post hoc comparisons
2.0 Mean	Nasal	1.0 [0.6, 2.5]	3.5 [0.8, 6.4]	0.68 [0.4, 1.4]	2.3 [1.4, 4.9]	0.13	
	Mandibular	2.8 [2.1, 8.1]	6.9 [5.4, 10.2]	–	3.5 [1.4, 13.7]	0.56	
	*p* Value	0.03			0.16	–	
1.5 Mean	Nasal	2.8 [0.8, 3.4]	4.1 [2.9, 7.4]	1.4 [0.4, 2.5]	3.5 [2.4, 5.1]	**0.008**	
	Mandibular	6.8 [4.5, 10.5]	9.9 [6.1, 11.9]	–	5.1 [2.4, 9.1]	0.27	
	*p* Value	**0.01**	**0.01**	–	0.16	–	
1.0 Mean	Nasal	2.4 [1.2, 4.4]	4.4 [2.5, 7.1]	1.5 [1.2, 3.2]	3.7 [3.0, 6.4]	0.03	
	Mandibular	8.9 [5.1, 10.2]	10.5 [6.3, 19.2]	–	7.2 [4.7, 9.0]	0.10	
	*p* Value	**0.01**	0.02	–	0.26	–	
0.5 Mean	Nasal	3.9 [2.1, 6.1]	4.2 [3.2, 11.1]	1.4 [1.2, 4.3]	5.4[4.1, 8.9]	**0.01**	Drop < Flash, *p* = 0.01^a^
	Mandibular	11.6 [8.6, 14.4]	12.8 [9.5, 15.6]	–	8.9 [6.3, 14.7]	0.36	
	*p* Value	0.03	**0.01**	–	0.16	–	
0.0 Mean	Nasal	5.8 [3.5, 8.9]	5.2 [4.3, 14.7]	2.7 [1.9, 6.6]	10.3 [5.6, 13.4]	**0.006**	Drop < Flash, *p* = 0.005^b^
	Mandibular	13.6 [9.5, 16.0]	16.2 [9.3, 18.1]	–	12.3 [9.3, 18.1]	0.65	
	*p* Value	**0.01**	**0.01**	–	0.16	–	
2.0 Maximal	Nasal	2.7 [1.4, 5.7]	6.6 [3.1, 9.7]	1.7 [0.9, 2.0]	5.9 [3.3, 9.1]	**<0.001**	Drop < Flash, *p* = 0.001^c^ Drop < Swedish, *p* = 0.001^d^
	Mandibular	5.6 [3.7, 11.4]	9.5 [5.5, 16.2]	–	4.3 [2.1, 17.4]	0.60	
	*p* Value	**0.01**	0.11	–	0.33	–	
1.5 Maximal	Nasal	4.7 [2.7, 6.9]	8.3 [4.8, 12.1]	2.5 [1.7, 5.3]	7.1 [4.1, 11.6]	**<0.001**	Drop < Flash, *p* ≤ 0.001^e^ Drop < Swedish, *p* ≤ 0.001^f^
	Mandibular	11.6 [6.6, 15.3]	14.9 [10.6, 24.6]	–	7.4 [3.1, 18.1]	–	
	*p* Value	**0.002**	0.02	–	0.14	–	
1.0 Maximal	Nasal	7.7 [4.1, 9.5]	8.6 [6.4, 14.7]	4.2 [3.4, 6.5]	10.6 [8.3, 13.2]	**<0.001**	Drop < Flash, *p* ≤ 0.001^g^ Drop < Swedish, *p* ≤ 0.001^h^
	Mandibular	13.8 [9.5, 18.6]	21.4 [13.3, 28.1]	–	11.2 [9.0, 14.5]	0.16	
	*p* Value	**0.003**	**0.006**	–	0.12	–	
0.5 Maximal	Nasal	9.8 [6.0., 14.3]	11.6 [7.7, 15.2]	8.0 [5.7, 10.1]	13.3 [9.3, 15.4]	**0.005**	Drop < Flash, *p* = 0.003^i^
	Mandibular	17.9 [13.2, 23.4]	25.6 [17.5, 36.8]	–	14.0 [10.3, 17.3	0.06	
	*p* Value	**0.002**	**<0.001**	–	0.12	–	
0.0 Maximal	Nasal	9.9 [7.7, 14.7]	12.1 [9.9, 13.5]	11.3 [8.6, 12.6]	14.4 [11.4, 16.8]	**0.003**	Drop < Flash, *p* = 0.006^j^ Cavesson < Flash, *p* = 0.01^k^
	Mandibular	18.4 [10.4, 22.4]	32.9 [19.5, 39.3]	–	15.6 [14.5, 25.6]	0.11	
	*p* Value	**0.002**	**0.002**	–	0.19	–	
2.0 Minimal	Nasal	0.2 [0.0, 0.5]	1.1 [0.2, 4.1]	0.1 [0.0, 0.9]	0.9 [0.1, 1.2]	**0.01**	*
	Mandibular	1.5 [0.9, 6.9]	4.3 [2.9, 7.8]	–	2.8 [0.7, 6.9]	0.42	
	*p* Value	**0.001**	**0.01**	–	0.02	–	
1.5 Minimal	Nasal	1.2 [0.3, 1.6]	2.6 [0.1, 4.0]	0.6 [0.4, 0.8]	1.8 [0.6, 2.4]	**<0.001**	Drop < Flash, *p* = 0.002^l^ Drop < Swedish, *p* = 0.003^m^
	Mandibular	4.8 [3.3, 8.5]	9.2 [4.5, 12.9]	–	3.8 [1.7, 8.4]	0.13	
	*p* Value	**<0.001**	0.03	–	**0.01**	–	
1.0 Minimal	Nasal	1.8 [0.8, 3.5]	3.1 [0.6, 7.3]	1.3 [0.6, 1.7]	4.2 [1.6, 6.0]	**0.002**	Drop < Swedish, *p* = 0.003^n^ Drop < Flash, *p* = 0.002°
	Mandibular	7.1 [4.4, 10.3]	15.0 [3.9, 19.4]	–	5.9 [2.8, 12.4]	0.23	
	*p* Value	**0.003**	**0.01**	–	0.05	–	
0.5 Minimal	Nasal	3.4 [2.1, 5.5]	4.8 [2.7, 9.0]	2.7 [1.3, 3.7]	5.4 [2.5, 7.9]	0.02	
	Mandibular	11.1 [8.8, 13.1]	18.1 [9.9, 21.8]	–	9.2 [5.1, 11.6]	0.04	
	*p* Value	**<0.001**	**0.01**	**–**	0.03	–	
0.0 Minimal	Nasal	5.1 [2.9, 7.5]	5.4 [2.3, 11.3]	4.8 [3.6, 5.8]	7.8 [4.8, 11.3]	0.09	
	Mandibular	11.9 [8.3, 14.8]	26.0 [17.8, 28.0]	–	13.8 [10.9, 17.2]	**0.004**	Cavesson < Swedish, *p* = 0.003^p^
	*p* Value	**<0.001**	0.02	–	0.02	–	
2.0 Force (nasal + mandibular)	Total	37.7 [16.6, 64.8]	73.3 [43.7, 113.9]	–	42.4 [22.4, 116.0]	0.56	
1.5 Force (nasal + mandibular)	Total	65.5 [44.6, 83.5]	84.8 [57.5, 116.3]	–	66.6 [31.1, 79.3]	0.56	
1.0 Force (nasal + mandibular)	Total	74.1 [44.1, 107.2]	108.1 [47.3, 121.2]	–	75.2 [63.4, 102.5]	0.27	
0.5 Force (nasal + mandibular)	Total	98.9 [87.9, 111.7]	111.0 [87.6, 181.6]	–	110.4 [81.9, 135.5]	0.56	
0.0 Force (nasal + mandibular)	Total	109.2 [82.0, 159.1]	138.4 [74.9, 233.1]	–	156.4 [114.8, 215.2]	0.36	

*Note*: Bold values indicate significant differences (*p* ≤ 0.01) (*indicates no post hoc differences found) between conditions, and nasal and mandibular pressures. Shaded boxes indicate no statistical test was performed. Superscripts (a–p) represented by the same character indicate post hoc differences between two nosebands; boxplots can be viewed in Figure 5.

Differences in maximal nasal pressure were found between noseband types when adjusted to 2.0, 1.5 and 1.0 finger tightness (all *p* ≤ 0.001), with the Drop noseband having lower maximal values than the Flash and Swedish (crank) noseband (all *p* ≤ 0.001). When adjusted to 0.5 and 0.0 finger tightness, differences were found (*p* = 0.005 and *p* = 0.003, respectively), with the Drop noseband having lower maximal nasal pressure values than the Flash (*p* = 0.003) at 0.5 finger tightness. At 0.0 fingers, the maximal nasal pressure was less for the Drop and Cavesson nosebands than the Flash (*p* = 0.006 and *p* = 0.01, respectively). No differences were found between the remaining noseband tightness conditions or noseband types (*p* > 0.06). For all tightness adjustments, when fitted with a Cavesson noseband and at 1.0, 0.5 and 0.0 finger tightness for the Swedish (crank) noseband, maximum pressures were higher for the mandible compared with nasal pressures (all *p* ≤ 0.01). No differences in maximal pressures between the mandibular and nasal bones were found for the Flash noseband (*p* ≥ 0.12) (Table 3; Figure 5).

Differences in minimal nasal pressure were found between nosebands when adjusted to 1.5 fingers (*p* ≤ 0.001), with the Drop noseband having lower minimal nasal pressure values than the Flash and Swedish (crank) noseband (*p* = 0.002 and *p* = 0.003, respectively); 1.0 finger (*p* = 0.002) with the Drop noseband having lower minimal values than the Flash and Swedish (crank) noseband (*p* = 0.002 and *p* = 0.003, respectively). At 0.0 finger tightness (*p* = 0.004), the Cavesson had lower minimal mandibular pressure than the Swedish (crank) (*p* = 0.003). No differences were found between the remaining noseband tightness conditions or noseband types (*p* > 0.02). For all tightness adjustments for the Cavesson noseband, and at 2.0, 1.0 and 0.5 finger tightness for the Swedish (crank) noseband and at 1.5 finger tightness for the flash noseband, minimal pressure values were higher for the mandible compared with nasal pressures (all *p* ≤ 0.01) (Table 3; Figure 5).

### 3.4 Total noseband force (N) for four noseband types adjusted from 2.0 fingers to 0.0 fingers tightness equivalents

No differences were found in total force between the Cavesson, Swedish and Flash nosebands at 2.0, 1.5, 1.0, 0.5 or 0.0 finger tightness equivalents (*p* = 0.6, *p* = 0.6, *p* = 0.3, *p* = 0.6 and *p* = 0.4, respectively) (Table 3).

## 4 DISCUSSION

This study compares mean, maximal and minimal noseband pressures over the nasal and mandibular bones for different types of nosebands adjusted to different tightness levels. The motivation to perform this work was to address appropriate noseband tightness during training and competitions. Our first hypothesis that nasal and mandibular pressures increase with noseband tightness was supported by the tighter noseband conditions. There were no significant differences in pressures between 2.0 and 1.5 finger tightness, but pressures were incrementally greater for nosebands set at 1.0, 0.5 or 0.0 finger tightness. Thus, tightening the noseband from 2.0 fingers to 1.5 fingers did not significantly increase pressure on the horse's nasal bone and mandibular rami, but nosebands with less laxity were associated with increasing levels of pressure.

**FIGURE 6:**
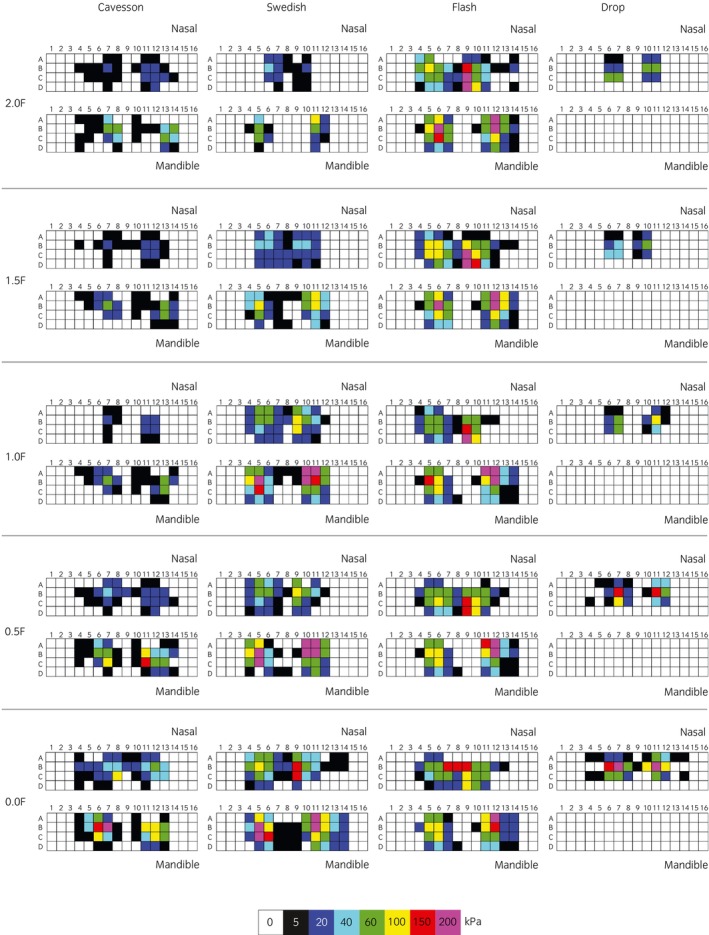
Mean nasal and mandibular pressure scans recorded from one horse fitted with a Cavesson, Swedish, Flash and Drop noseband (left to right columns) at tightness levels of 2.0, 1.5, 1.0, 0.5 and 0.0 finger tightness (rows from upper to lower) when ridden in rising trot. Colour calibration shown at the foot of the scans. For the Drop noseband, only the nasal mat was used because the position of the ventral strap in the chin groove was not amenable to mat placement.

Our second hypothesis that pressure magnitude differs between noseband types was also supported. The Drop noseband had the lowest absolute nasal pressures and significantly lower values than the other nosebands. Based on our experience in the examination of horses and description in anatomical texts, the nasal contour beneath the Drop noseband seems less likely to have a central depression or bridging on the lateral sides, in contrast to the contour at the level of the Cavesson or Swedish (crank) noseband position.^20^ Even so, the pressure scans (Figure 6) indicate a relatively narrow loaded area, which is likely to be influenced by the rostral extent of the nasal bone at this level.

For data where all noseband tightness was pooled (Table 1; Figure 1), differences between noseband types were found, with the Drop and Cavesson having a lower mean and minimal dorsal nasal pressure than the Swedish (crank) and the Flash noseband. Maximal mandibular pressures were lower for the Flash noseband compared with the Swedish noseband, whilst minimal mandibular pressure was lower for the Cavesson and Flash noseband compared with the Swedish (crank). The pooled data would indicate that the pressures were higher for the Swedish noseband; however, when comparing nosebands adjusted to the same tightness (Table 3; Figure 5), there were minimal differences between the Swedish and Cavesson nosebands, the exception being a higher minimal mandibular pressure on the Swedish (crank) noseband with 0.0 fingers adjustment. This contradicts previous publications referring to the Swedish (crank) noseband as a ‘jaw clamping’ noseband.^2^ This may be because of the previous assumption that there is a leverage effect on the noseband fastening. However, this fails to take into account one D‐ring being used to adjust and stabilise the ventral pad position and the other acting alone as a pulley system.

Our third hypothesis that mandibular pressures would exceed nasal pressures was accepted at all tightness levels for the Cavesson, Swedish and Flash nosebands. This confirms the findings of a modelling study^4^ that predicted noseband pressure would be 1.33 times higher over the more convex bony curvatures of the mandibular rami than the outer edge of the nasal bones. Maximal nasal pressure for the tightest (0.0 finger tightness) Swedish (crank) noseband in our study is lower than that reported by Murray et al. for a Swedish (crank) noseband with or without a modification to alleviate pressure on the lateral aspect of the nasal bones.^8^ It is possible that the differences in values recorded between these studies relate to differences in noseband padding or bridle design, bridle fit, anatomical shape of the horses' heads in the different studies, differences in rider rein tension or bit design or the kinematics of the horses in the different studies.

Determining a threshold at which pressure induces pain and discomfort is biologically complex, depending on the interaction between the nature, duration and magnitude of pressure with the biological response. Maximal pressure values of 53.3 kPa and mean pressure of 29.7 kPa reported for the lateral aspect of the horse's withers have been associated with epaxial musculature hypertonicity and localised areas of dry skin due to sweat gland compression.^21^ As a guide, saddle maximal and mean pressures should not exceed 30 and 11 kPa, respectively.^22^ However, higher pressures under saddles have been reported in dressage,^23^ jumping^24^ and racehorses^25^ without any hypertonicity of the epaxial musculature or physiological responses indicative of pain or dysfunction. It is difficult to directly compare these thresholds, which were obtained for less curved locations with greater muscle coverage, fewer anatomical prominences, and less movement of the anatomical prominences, with the locations in the head being evaluated in this study.

**FIGURE 7:**
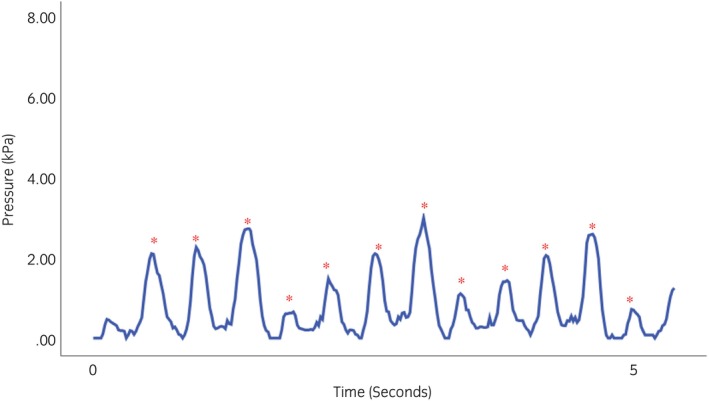
Mean dorsal nasal pressure (kPa) beneath a Cavesson noseband adjusted to 2.0 finger equivalent tightness. The graph illustrates the cyclical nature of noseband pressure taken from one horse when passing through the experimental track on the left rein. The * represents the maxima, which occurs during mid‐stance.

Pressure applied to the skin surface penetrates the underlying tissues until it reaches the bone, where pressure is higher than at the skin surface.^26^ The mean capillary pressure in humans is approximately 3.33 kPa.^27^ When an external pressure continuously exceeds 4.26 kPa, the blood vessels are occluded. Noseband pressures, however, are transient and change with head, neck and limb movements.^8^ In the current study, with the noseband adjusted to 0.0 finger tightness, mean nasal and mandibular pressures were 6.4 and 14.6 kPa, while at 2.0 finger tightness, they were 1.6 and 5.4 kPa (Table 2). Some authors have likened a tight noseband to a tourniquet,^3^ but this analogy is refuted by the cyclic nature of the noseband pressure–time curve (Figure 7) and reported variation in pressures during the stride.^8^ A tourniquet is based on applying continuous high pressure to occlude underlying vessels.^28^ The physiological effects of pressure depend on both the magnitude and time for which it is applied, with an intermittent pattern of pressure increases and decreases potentially being of less concern than continuous high pressure.

Study limitations include the fact that although nosebands were of the same weight, contact area and design, they were not identical. While this study has quantified nasal and mandibular pressures under four types of nosebands, there are many variations in design and fit that were not tested. Due to the size of the pressure mat, pressure values for the lower band for the Drop and Flash noseband were not investigated, and the position of both needs to be considered in relation to the anatomy and underlying structures. Horse‐rider variation is always present, but we attempted to minimise variation by using high‐level dressage horses and riders; therefore, applying these findings to less skilled horses and riders should be done so with caution. Future work should investigate noseband pressures in less skilled horse/rider combinations. To comply with ethical requirements, the order in which the tightness levels were sequentially increased (2.0 finger to 0.0 finger tightness equivalents) was not randomised. Adjustment tighter than 0.0 fingers is characterised by the visual compression of the soft tissues. Our study defined 0.0 finger‐equivalent tightness as the inability to admit the taper gauge beneath the noseband and with no compression of the soft tissues; therefore, applying these findings to nosebands that are tighter than 0.0 fingers should be applied with caution.

Whilst this is a positive step forward in advancing our understanding of the effect that the noseband has on the horse, other factors should also be considered, including the horses' behavioural response to noseband tightening. In a group of horses naïve to wearing a double bridle and Swedish noseband, an increase in eye temperature was reported,^29^ although in high‐level dressage horses, when a Cavesson noseband was adjusted from 2.0 to 0.0 finger tightness, no difference in eye temperature or blink rate was reported (Clayton et al., 2024, unpublished data) when standing and chewing a treat. It might, therefore, be of value to consider future studies to compare equine behavioural and physiological parameters between different noseband tightness levels or noseband types under different ridden conditions.

## 5 CONCLUSION

In equestrian sports, rules are necessary to safeguard the horse's welfare and safety and evidence‐based data are needed to guide regulatory bodies. At the present time, noseband tightness is a controversial area^12^, ^13^ which was the motivation to perform this study, to provide evidence to guide the development of policies regarding noseband adjustment. Based on our findings, tightening the noseband from 2.0 fingers to 1.5 fingers did not significantly increase pressure on the horse's head and was not within a range at which pain or tissue damage would be expected to occur, which may be useful when considering rules and regulations. In a group of high‐level dressage horses ridden in trot on straight lines, mean, minimal and maximal nasal and mandibular pressures increased with noseband tightness, with 1.0 finger laxity or less being associated with significantly and incrementally higher pressures than 1.5 or two fingers tightness. The Drop noseband had the lowest nasal pressures, which was thought to be due not only to its design but also to its location. When reviewing individual noseband tightness adjustments, no differences were seen between the Cavesson and Swedish (crank) at all tightness levels, with the exception of minimal pressure at 0.0 finger tightness. For all noseband types, noseband pressures were higher for the mandible, further work is needed to determine if the magnitude of pressure can be reduced with noseband modifications and padding, particularly beneath the noseband crossing under the mandibles.

## FUNDING INFORMATION

This research was funded by World Horse Welfare, Canadian Sport Horse Association, Hartpury University, British Equestrian Federation and The Worshipful Company of Saddlers.

## CONFLICT OF INTEREST STATEMENT

The authors declare no conflict of interest.

## AUTHOR CONTRIBUTIONS

**Russell MacKechnie‐Guire:** Conceptualization; data curation; formal analysis; visualization; writing – original draft; methodology; investigation; project administration; writing – review and editing; resources; funding acquisition; software. **Rachel Murray:** Conceptualization; data curation; formal analysis; visualization; writing – original draft; investigation; methodology; supervision; writing – review and editing; resources. **Jane M. Williams:** Formal analysis; data curation; conceptualization; project administration; writing – review and editing; funding acquisition; resources; methodology. **Jane Nixon:** Conceptualization; methodology; writing – review and editing; resources; investigation. **Mark Fisher:** Methodology; investigation; writing – review and editing; conceptualization. **Diane Fisher:** Conceptualization; methodology; investigation; writing – review and editing. **Vicki Walker:** Conceptualization; data curation; formal analysis; writing – review and editing; methodology; investigation. **Hilary M. Clayton:** Conceptualization; data curation; formal analysis; visualization; writing – original draft; methodology; investigation; writing – review and editing; funding acquisition; software.

## DATA INTEGRITY STATEMENT

Russell MacKechnie‐Guire, Rachel Murray, Jane Williams, Victoria Walker and Hilary Clayton.

## ETHICAL ANIMAL RESEARCH

The study was approved by Hartpury University's Ethics Committee: URN 2021‐105.

## INFORMED CONSENT

Informed, written consent was obtained from riders and horse owners.

## Data Availability

The data that support findings of this study are available from the corresponding author upon reasonable request: Open sharing exemption granted by the editor due to lack of provision in the owner informed consent process.
